# Pericardial Cyst in Birt-Hogg-Dubé Syndrome: An Unexpected Discovery

**DOI:** 10.7759/cureus.41728

**Published:** 2023-07-11

**Authors:** Gagandeep Singh Arora, Sudeshna Ghosh, Jarmanjeet Singh

**Affiliations:** 1 Internal Medicine, University of California, Riverside, School of Medicine, Riverside, USA; 2 Cardiology, University of California, Riverside, School of Medicine, Riverside, USA

**Keywords:** lower lobe predominance, cystic lung disease, genetic syndromes, pneumothoraces, nonobstructive coronary artery disease, coronary artery disease, pericardial cyst, elderly patient, chest discomfort, birt-hogg-dubé syndrome

## Abstract

Birt-Hogg-Dubé syndrome (BHD) is a genetic disorder typically characterized by pulmonary cysts, cutaneous fibrofolliculomas, and renal tumors. We report a case of an 87-year-old male patient with a known diagnosis of BHD and a large pericardial cyst who presented to the emergency room with chest pain. BHD is classically associated with pulmonary cysts and not pericardial cysts. In this report, we highlight the potential of pericardial cysts to independently cause retrosternal pain resembling angina, while also mentioning that BHD too can cause chest pain through the rupture of a pulmonary cyst leading to spontaneous pneumothorax. In our case, coronary angiography revealed non-obstructive coronary arteries, so the cause of chest pain was attributed to myocardial infarction with non-obstructive coronary arteries (MINOCA). Atypical causes of chest pain should be considered, especially in patients with diagnosed genetic syndromes.

## Introduction

Chest pain is a common presenting symptom in the Emergency Room (ER) that can caused by a variety of conditions, including but not limited to, heart-related issues like angina or heart attack, pulmonary conditions like pneumonia or pulmonary embolism, gastrointestinal problems like gastroesophageal reflux disease (GERD), musculoskeletal issues such as costochondritis, and even psychological factors like panic disorders and infectious causes like shingles [1]. Chest pain due to coronary artery disease is usually ruled out prior to considering other causes as missing this could lead to a fatal outcome. The patient’s history of classic chest pain is usually a retrosternal squeezing pain or tightness radiating to the left arm, triggered by exertion or stress, relieved with rest, and accompanied by shortness of breath and sweating. In the ER, troponin levels and EKG provide objective evidence of myocardial ischemia/myocardial infarction [1]. While an echocardiogram provides an estimation of the function of the heart, coronary angiography provides conclusive evidence for identifying coronary artery disease. 

A pericardial cyst is a rare, congenital condition characterized by a fluid-filled sac on the pericardium, which can cause symptoms like chest pain, shortness of breath, and coughing due to the cyst exerting pressure on nearby structures [2]. While often asymptomatic, serious complications can occur such as cyst rupture or cardiac tamponade, warranting imaging studies for confirmation and a potential range of treatments from watchful waiting to surgical removal based on the cyst's size and symptom severity [2].

Birt-Hogg-Dubé syndrome (BHD), a rare genetic disorder, typically manifests through skin abnormalities such as fibrofolliculomas, trichodiscomas, and acrochordons, as well as multiple lung cysts leading to potential spontaneous pneumothorax characterized by sudden chest pain, shortness of breath, and occasional cough [3]. Further, the syndrome significantly increases the risk of both benign and malignant kidney tumors, often asymptomatic in early stages but may present as blood in urine, back or side pain, and a noticeable mass as they grow, alongside possible general symptoms like fatigue or weight loss, emphasizing the need for regular health monitoring for those affected [4].

BHD is classically associated with pulmonary cysts and not pericardial cysts. In this report, we present a rare confluence of the two occurring together in the same patient. We wish to highlight that pericardial cysts can, by themselves, cause retrosternal pain, which can mimic angina, and BHD can cause chest pain via the rupture of a pulmonary cyst, resulting in spontaneous pneumothorax. However, in our patient, chest pain was likely due to nonobstructive coronary artery disease. 

## Case presentation

An 87-year-old gentleman with a medical history of hypothyroidism and low-grade prostate cancer, for which he had not received surgery or chemoradiation, was referred from local community hospital to the ER with chest pain. He was a retired teacher who had never smoked or consumed alcohol.

This distressing episode began two nights prior to presentation when he was suddenly awoken from sleep at 2 AM with a retrosternal chest pain, which he rated as 6/10 initially, but gradually intensified to an 8/10. The pain did not radiate to the neck, jaw, shoulder, or arm. It did not exacerbate upon exertion or physical activity and was not associated with shortness of breath, palpitations, nausea, vomiting, diaphoresis, dizziness, syncope, cough, or fever.

The subsequent afternoon, while visiting his daughter's residence, he experienced a similar pain episode. His daughter, a professional nurse, measured his vital signs, which according to the patient, were within normal parameters. However, considering the recurrence of the pain, he decided to seek emergency medical attention. It is noteworthy that he had no prior episodes of similar pain. His only home medication was levothyroxine, taken for hypothyroidism.

Upon arrival at the local community hospital's ER, he was pain-free. Despite this, a prophylactic dose of sublingual nitroglycerin and aspirin (325 milligrams) were administered. An electrocardiogram (EKG) revealed bradycardia with sinus rhythm, while his high sensitivity troponins were found to be elevated at 1.230 nanograms/liter (ng/L) (reference range 0 and 0.04 ng/mL). Given his advanced age and the elevated troponins, he was referred to our hospital for further evaluation.

At our facility, standard blood work, a chest X-Ray, EKG, and a second high-sensitivity troponin test were conducted. A comprehensive metabolic panel, including thyroid-stimulating hormone (TSH) was within normal limits. The chest X-ray revealed mild patchy lung opacities in the mid aspect of the right lung, possibly due to pleural thickening, and some calcification at the right lung base.

The EKG reiterated the sinus bradycardia and his troponin levels had risen further to 2.330 ng/L. Consequently, Cardiology was consulted, and it was decided to start the patient on a heparin drip and schedule a left heart catheterization for the following morning. Concurrently, the ER physician ordered an echocardiogram and a CT scan of the chest with intravenous contrast.

A detailed history unveiled a prior episode of spontaneous pneumothorax 50 years ago, treated with a right-sided chest tube. Various family members, including his father and brothers, also had past episodes of spontaneous pneumothorax. The patient had been prescribed an albuterol inhaler by a pulmonologist for occasional exertional dyspnea, which he had stopped using. A cardiothoracic surgeon consultation six years prior had identified a 'cyst' adjacent to his heart, but it was deemed non-symptomatic and did not warrant surgical intervention.

**Figure 1:**
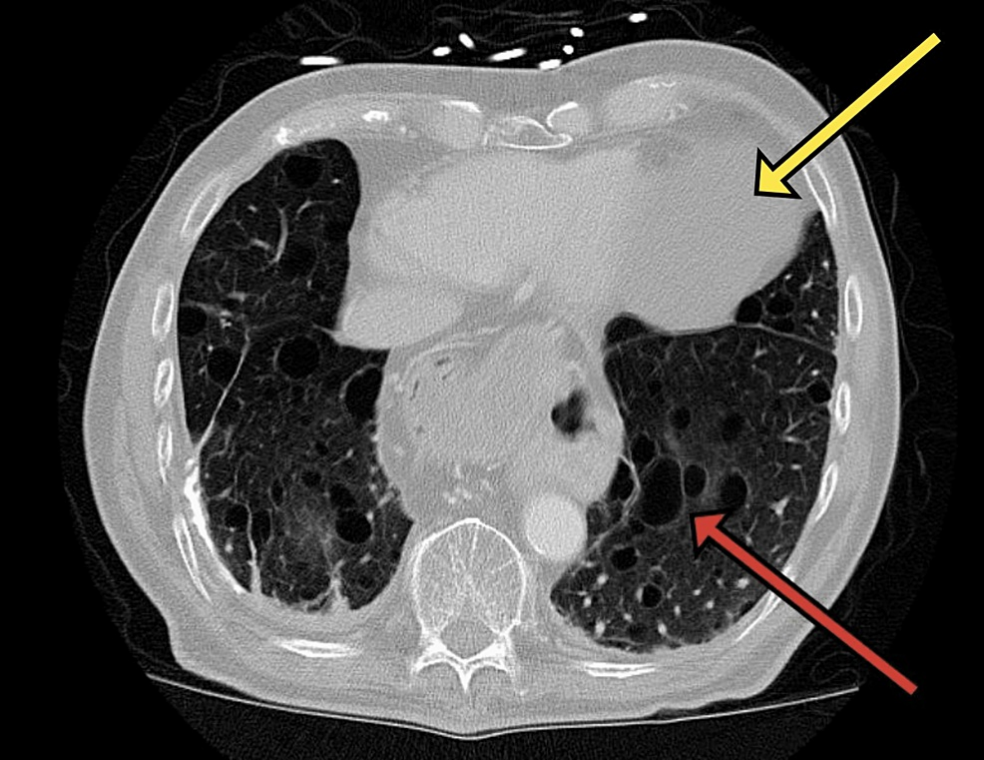
CT scan of the chest (transverse section of lung) showing one of the larger cysts (red arrow) in the background of cystic lung disease, and the pericardial cyst (yellow arrow).

**Figure 2:**
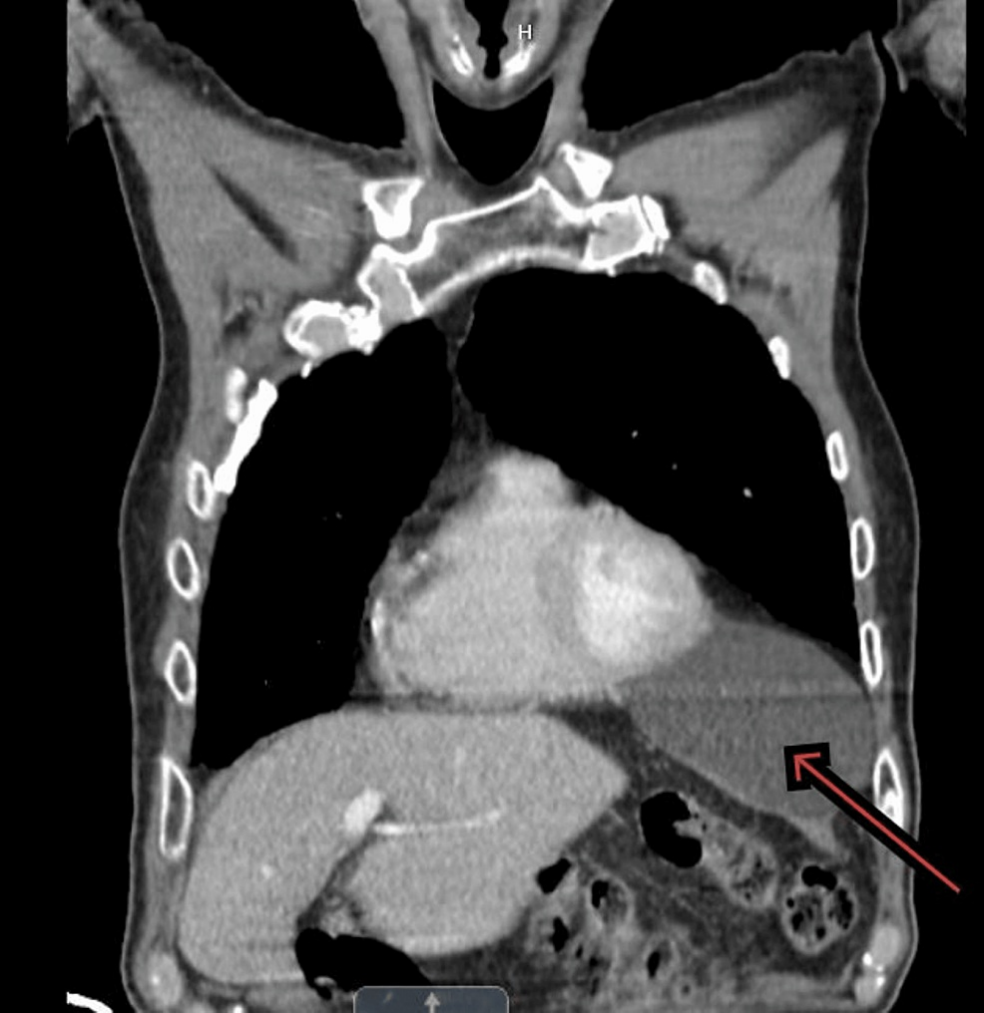
CT scan of chest in coronal section showing a big pericardial cyst (red arrow)

**Figure 3:**
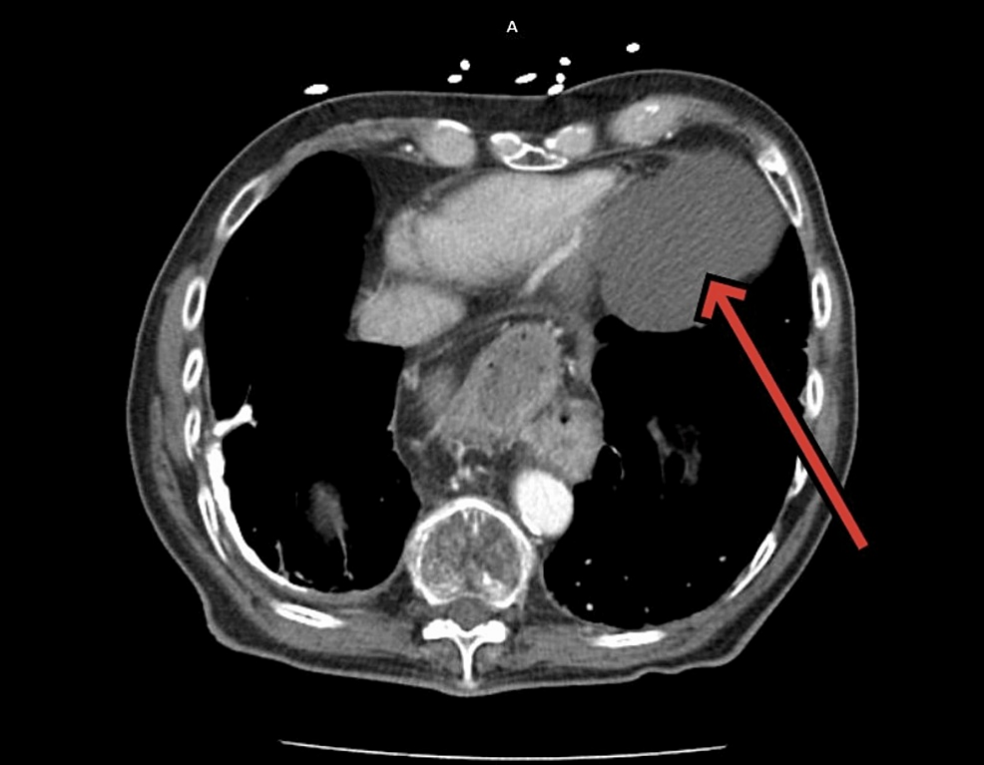
CT scan in transverse section showing pericardial cyst (red arrow)

The CT chest report confirmed no evidence of pulmonary malignancy. It revealed prominent parenchyma demonstrating cystic structures which had lower lobe predominance consistent with cystic lung disease (Figure 1) and at the left anterior cardiophrenic margin, there was a minimal complex fluid attenuation cyst measuring 5.8 x 8.1 x 6.0 cm, consistent with a benign pericardial cyst (Figure 2 and Figure 3). A large paraesophageal hernia was also seen. The report suggested that this pattern might be indicative of lymphangioleiomyomatosis.

Further information from the patient's daughter revealed that he had been diagnosed with BHD, a genetic condition that runs in their family, six years ago after undergoing genetic testing. His brother too was affected by this syndrome.

The following morning, the patient's bradycardia resolved spontaneously. The echocardiogram report stated that the left ventricle cavity size and wall thickness were normal, with a normal systolic function and an ejection fraction of 60%. No regional wall motion abnormalities were noted.

The angiogram displayed a right dominant circulation, with minimal luminal irregularities in the left main vessel. The left anterior descending and left circumflex arteries were of normal caliber but had mild to moderate diffuse disease. The right coronary artery showed similar diffuse disease. It also gave rise to the right posterior descending and posterolateral branches.

The cardiology team concluded that the patient likely had experienced a myocardial infarction with non-obstructive coronary arteries (MINOCA). They hypothesized that the observed bradycardia was a vagal response to the pain or an ischemic response to right coronary artery disease. As the angiogram did not indicate the need for a stent, medical management was advised. The patient was discharged on a regimen of aspirin (81 mg orally once a day), clopidogrel (75 mg orally once a day), atorvastatin (40 mg orally once a day), isosorbide mononitrate extended-release (30 mg orally once a day), and amlodipine (5 mg orally once a day) for its anti-anginal effect. A follow-up visit with the cardiology clinic was arranged before discharge.

## Discussion

Pericardial cysts, often incidental findings on routine chest imaging, are a rare congenital anomaly with an incidence of approximately one in 100,000 persons [5]. They comprise around 33% of all mediastinal cysts and 7% of mediastinal masses [5]. These cysts are generally asymptomatic but may become symptomatic when they compress adjacent structures, causing symptoms like retrosternal pain, cardiac dysfunction, and respiratory issues such as recurrent infections and dyspnea [5]. Rare complications can arise due to compression of adjacent structures, cyst infection, compression of the superior vena cava, and hemorrhage into the pericardial space, potentially leading to tamponade and death [6].

Different diagnostic modalities aid in the evaluation of pericardial cysts. CT scans offer clear and sharp images of the cysts and are advantageous due to their lack of motion artifacts and short acquisition time [2]. Echocardiography, a safe and cost-effective method, is also useful for follow-up and image-guided percutaneous aspiration, despite some limitations such as limited windows and a narrow field of view [2]. Cardiac MRI, though time-consuming and expensive, provides excellent soft tissue visualization [2].

The primary treatment approach for pericardial cysts is conservative, entailing regular monitoring with echocardiography [5]. However, symptomatic or enlarging cysts necessitate surgical intervention, with options including percutaneous aspiration, ablation/ethanol sclerosis, or surgical resection via thoracotomy, sternotomy, video-assisted thoracic surgery (VATS), or mediastinoscopy [5]. Surgery is recommended in symptomatic patients, particularly those with large cysts, radiological signs of compression, impending compression to vital structures, or potential for malignant transformation [2]. A patient diagnosed with a pericardial cyst will be under serial echocardiography follow-up to detect any compressive effect of the cyst on vital structures, hemorrhage, infection, or cyst rupture [2].

On the other hand, BHD caused by germline mutations in the folliculin (*FLCN*) gene, presents with noncancerous cutaneous lesions, multiple pulmonary cysts, spontaneous pneumothoraces, and varied renal tumors [3,7]. A diagnosis of BHD should be considered based on personal and/or family history of these manifestations. A definitive diagnosis is made by genetic testing demonstrating *FLCN* germline mutation [3].

Lung involvement is observed in 80% of BHD patients, with spontaneous pneumothorax as the presenting feature in 25% of patients [8]. The development of spontaneous pneumothorax in BHD may be due to the stretch hypothesis, which proposes that cysts in the lungs of BHD patients develop due to stretch-induced stress at regions with weaker cell-cell adhesion forces [9]. Surveillance for renal tumors is advised in BHD patients with abdominal MRI or CT from the age of 21, given that ultrasound may fail to detect small masses [4]. When identified, nephron-sparing surgery is recommended for renal cancers larger than 3 cm [4]. Therefore, identifying pathogenic *FLCN* germline mutation is crucial for definitive diagnosis, prognosis, and management, including screening and family counseling [7].

In summary, BHD Is associated with pulmonary cysts and the presence of pericardial cysts is rare. To the best of our knowledge, it has been reported once earlier by Sharma et al [10]. Both pericardial cysts and BHD present unique challenges in their diagnosis and management. The interplay of these two rare conditions in a single patient might have implications for their management, with a focus on comprehensive genetic counseling and regular monitoring for possible complications. Despite this, further research is needed to identify the potential association between these two conditions. 

Although pericardial cysts can cause chest pain, our patient's chest pain was likely due to non-obstructive coronary artery disease since he had elevated troponins at presentation. Further, our cardiology team decided that no acute intervention was needed for the cyst and asked him to follow up as an outpatient to monitor its size.

This case emphasizes the necessity of considering non-standard causes of chest discomfort in elderly patients, especially those with identified genetic syndromes.

## Conclusions

This case report highlights a rare association between BHD and pericardial cysts and their potential contribution to chest pain. While coronary artery disease is a common cause of chest pain, alternative explanations should be considered when angiography does not reveal obstructive lesions. The presence of BHD and pericardial cysts in this patient emphasizes the importance of comprehensive genetic counseling. While a patient with BHD is monitored for renal cell carcinoma via renal ultrasound, monitoring of large pericardial cysts is done via echocardiogram and CT scan. Pericardial cysts can cause retrosternal pain similar to angina, and BHD can also lead to chest pain through pulmonary cyst rupture, causing spontaneous pneumothorax. Atypical causes of chest pain should be considered in patients with known genetic conditions.
